# Malaria Microscopy Competency in the Subnational Verification, China: Implications for Malaria Elimination and the Prevention of Malaria Reestablishment

**DOI:** 10.1155/2022/8003845

**Published:** 2022-10-30

**Authors:** Mei Li, Fang Huang, Jianhai Yin, He Yan, Shuisen Zhou, Zhigui Xia

**Affiliations:** National Institute of Parasitic Diseases, Chinese Center for Disease Control and Prevention (Chinese Center for Tropical Diseases Research), NHC Key Laboratory of Parasite and Vector Biology, WHO Collaborating Center for Tropical Diseases, National Center for International Research on Tropical Diseases, Shanghai 200025, China

## Abstract

**Introduction:**

Qualified microscopy competency is a key indicator for certification of malaria elimination. To better prepare the country certification and identify the priorities that need improvement to prevent malaria reestablishment, microscopy competency at different levels were assessed in subnational verification of malaria elimination in China. *Methodology*. Microscopist representatives from centers for disease control and prevention (CDC)/institutes of parasitic diseases (IPD) and medical institutes for malaria diagnosis at the provincial and county levels in the subnational verification were analyzed. Specifically, five provincial microscopist representatives and ten county-level representatives were assessed in each of previously endemic provinces on qualitative identification (*Plasmodium* positive or negative) and *Plasmodium* species identification using standard slides from the National Malaria Diagnosis Reference Laboratory.

**Results:**

A total of 100 provincial-level representatives (60 from 42 CDCs/IPDs and 40 from 34 medical institutes) and 200 county-level representatives (61 from 41 CDCs and 139 from 118 medical institutes) were included. The qualitative accuracy was higher than 90% each (*P* = 0.137), but slides with low parasite density were easy to be misdiagnosed as negative. Furthermore, the accuracy of species identification was 80.0% and 83.6% in medical institutes and centers for disease control and prevention (CDCs) at the provincial level (*P* = 0.407) with relatively high misdiagnosis of *P. vivax* as *P. ovale* in the latter (16.2%) and 82.0% and 85.0% in medical institutes and CDCs at the county level (*P* = 0.330) for the identification of *P. falciparum* and non-*P. falciparum* with higher false-negative in medical institutions (*P* < 0.001).

**Conclusions:**

In conclusion, competent microscopy in subnational verification supported the quality in eliminating malaria in China, while the accurate identification of malaria parasites, especially slides with low parasite density still need to be improved through continuous diagnostic platform construction, continuous technological innovation, and targeted training to prevent reestablishment of malaria transmission.

## 1. Introduction

Malaria is a serious and sometimes fatal disease caused by *Plasmodium* parasites that are transmitted to people through the bites of infected female *Anopheles* mosquitoes. According to the World Malaria Report 2021 [1], nearly half of the world's population was at risk of malaria, there were 241 million cases of malaria and 627 000 malaria deaths in 2020, and the most vulnerable populations were still infants, children under 5 years of age, pregnant women, and those with low immunity. Nevertheless, more and more countries were certified as malaria-free or approaching to malaria elimination [2, 3].

A fully functional surveillance and response system that can prevent reestablishment of indigenous transmission in place is one of the necessary prerequisites for certification of a country's malaria-free status by the World Health Organization (WHO) [4], and a robust and sensitive case-based surveillance system with qualified competency of case confirmation and classification to detect malaria infections is the primary component [5, 6]. Although there are a variety of diagnostic methods for malaria parasites identification and speciation, malaria microscopy is still the gold standard method recommended by WHO [7], and the qualified microscopy competency is a key indicator for certification of malaria elimination [8].

In order to achieve the goal of malaria elimination, China has made continuous efforts in malaria diagnosis, especially since the implementation of the China Malaria Elimination Action Plan in 2010, which has achieved remarkable progresses in many aspects [9]. First, China has formed a tutor team with high-level competency for malaria microscopy certified by WHO through the WHO External Competency Assessment (ECA) of Malaria Microscopists courses organized in the country, and the tutors are from national and provincial laboratories distributed in all the provinces in Chinese mainland [10, 11]. Second, the national malaria diagnosis network in health institutions at all levels, and a China malaria diagnosis reference laboratory network with a quality management system based at the provincial CDCs (center for disease control and prevention)/IPDs (institute of parasitic diseases) in all the 24 historically malaria-endemic provinces and one nonendemic province, has been established and works well [9, 12]. Third, a series of quality assessment activities for malaria diagnostic capacity, mainly based on malaria microscopy, such as routine blood sample review level by level, and national/WHO external quality assessment programs, have been held continuously [9–11]. Finally, the national technique competition on the prevention and control of parasitic diseases including malaria (theoretical knowledge, blood film preparation, identification and quantification of *Plasmodium* species using light microscopy) has been held annually since 2011 but before the coronavirus disease 2019 (COVID-19) pandemic to continuously promote capacity building in CDCs/IPDs and medical institutions across the country [11, 13]. In addition, regular competency training and quality control were also held in in all parts of the country.

On 30 June, 2021, China was certified as malaria-free [14], and before the WHO certification, subnational verification of malaria elimination was carried out in the country to strengthen the surveillance and response system and better prepare the country for national certification [4]. Meanwhile, the assessment of microscopy competency at different levels in the areas being assessed was the key component of the subnational verification and played a decisive role. In the present study, the performance of microscopist representatives for malaria diagnosis from the provincial and county levels in the subnational verification was analyzed to assess the malaria microscopy competency supporting malaria elimination in China and identify the priorities that need improvement in response to the prevention of malaria reestablishment of transmission.

## 2. Methodology

### 2.1. Microscopist Representatives

A total of 15 representatives composed of 5 provincial-level and 10 county-level microscopists were required in the subnational verification to assess the malaria microscopy competency in each province. Generally, two countries and their municipalities per province will be included in the assessment. Moreover, the provincial-level representatives per province were from various institutions including the provincial CDC/IPD, or the provincial designated hospital for malaria diagnosis and treatment, or the hospital with relatively more malaria cases at provincial level, or the prefectural CDCs or hospitals with relatively more malaria cases. In addition, the county-level representatives were from two counties averagely, and at least one from the county CDC and a county hospital each per county must be included. All the microscopists were nominated by their institutions, respectively.

### 2.2. Structure of the Standard Blood Slides

In the assessment, twelve sets of standard blood slides were used, and the difficulty of each set was similar based on the blood film quality, the composition of negative and positive, the Plasmodium species and their density, etc. Meanwhile, the fairness, scientificity, and operability were also considered comprehensively. In detail, each set was consisted of 5 blood slides positive for a single species of *Plasmodium* (*P. falciparum*, *P. vivax*, *P. ovale*, or *P. malariae*) or negative for *Plasmodium*, from the National Malaria Diagnosis Reference Laboratory, and one piece positive of *P. falciparum* (602–263000 p/*μ*l) and one piece positive of *P. vivax* (1138–17570 p/*μ*l) with high density or with typical morphology were included in each set. Moreover, one piece positive of *P. falciparum* slide with low-density of 126–471 p/*μ*l and one negative slide (different degrees of difficulty) were also included in each set. The remaining one slide in each set was selected from two *P. vivax* slides (254 p/*μ*l, 264 p/*μ*l), two *P. ovale* slides (>2000 p/*μ*l), two negative slides, two *P. malariae* slides (1199 p/*μ*l, 1731 p/*μ*l) and four *P. falciparum* slides (408–953 p/*μ*l), respectively.

### 2.3. Slide Reading

The twelve sets of standard slides were divided into 2 groups with 6 sets each, and 3 sets from one group were randomly selected at the right moment of assessment in one province; then, one set was distributed to every five of the 15 representatives in a province and read according to the requirements in external assessment of the competence of national core group microscopists recommended by WHO [7]. In the other words, each microscopist reviewed one set of slides totally. The time limit for reading each slide was 10 minutes. The replacement of blood slides, invigilation and timing were implemented by 2 members from the national assessment expert group.

The 15 microscopists should identify the malaria parasite species on 5 pieces of blood slides individually, and provincial-level representatives need to make a qualitative (positive or negative) and species-specific identification of each slide, while county-level representatives need to make a qualitative identification and the differentiation between *Plasmodium falciparum* and non-*Plasmodium falciparum*.

### 2.4. Statistical Analysis

The results of qualitative and species identification were entered in Microsoft Excel 2010 and analyzed using IBM SPSS Statistics 20. Then, Pearson chi-square test or Fisher's exact test were used to compare the performance differences in different institutes at provincial and county levels at 0.05 level of statistical significance.

## 3. Results

A total of 100 provincial-level representatives and 200 county-level representatives were included. At the provincial level, 60 representatives were from 42 CDCs/IPDs and 40 representatives were from 34 medical institutes. At the county level, 61 representatives were from 41 CDCs and 139 representatives were from 118 medical institutes. Overall, the qualitative accuracy of malaria microscopy from provincial and county-level medical and CDC representatives was higher than 90%, respectively, and the accuracy of species identification was from 80.0% to 85.0%.

### 3.1. Malaria Microscopy Performance at the Provincial Level

Among the representatives from the provincial CDCs/IPDs, a total of 24 (40.0%) representatives responded correctly to all 5 blood slides in both qualitative and species identification, and other 23 (38.3%), 8 (13.3%), 4 (6.7%), and 1 (1.7%) representatives read correctly 4, 3, 2, and 1 piece of blood slides, respectively. In terms of qualitative identification, 43 (71.7%) representatives were all right of 5 slides, 15 (2.5%) were wrong with 1 piece, and 2 (3.3%) were wrong with 2 pieces, and no significance was found (*P*=0.196, Fisher's exact test) (Table 1). Among the wrong slides, except for 9 negative pieces were identified as *Plasmodium-*positive slides, the rest were 7 *P. falciparum*-positive and 3 *P. vivax*-positive slides which were qualitatively identified as negative, and all the ten slides are with very low (<500 p/*μ*l) parasite density, and no significance was found in the accuracy of species identification (*P*=0.248, Fisher's exact test), but the relatively high percentage of *P. vivax* was identified as *P. ovale* (16.2%, 12/74) (Table 2).

Similarly, a total of 14 (35.0%) representatives responded correctly to all 5 blood slides in qualitative and species identification, and other 16 (40.0%), 5 (12.5%), and 5 (12.5%) representatives read correctly 4, 3, and 2 pieces of blood slides, respectively, from the provincial medical institutes. In terms of qualitative identification, 31 (77.5%) representatives were all right of 5 slides, 7 (17.5%) were wrong with 1 piece, and 2 (5.0%) were wrong with 2 pieces, and no significance was found (*P*=0.158, Fisher's exact test) (Table 3). Among the wrong slides, except for 5 negative pieces were identified as *Plasmodium-*positive slides, the rest were 6 *P. falciparum*-positive slides were qualitatively identified as negative, and all the six slides are with very low (<500 p/*μ*l) parasite density, and no significance was found in the accuracy of species identification (*P*=0.975, Fisher's exact test) (Table 4).

### 3.2. Malaria Microscopy Performance at the County Level

A total of 21 (34.4%) representatives from county CDCs got correct results of 5 blood slides in qualitative and species identification, and other 21 (34.4%), 17 (27.9%), 1 (1.6%), and 1 (1.6%) representatives read correctly 4, 3, 2 and 1 slides, respectively. Among them, a total of 37 (60.7%) representatives were all right of 5 slides, and 6 (9.8%) and 18 (29.5%) representatives were wrong with 2 and 1 slides, respectively, in terms of qualitative identification. Furthermore, the *Plasmodium*-negative and *P. falciparum*-positive slides were more likely to be misdiagnosed (*P* = 0.001, Fisher's exact test) (Table 5). In addition to 11 negative slides, 19 *P. falciparum*-positive slides were erroneously classified as negative, among which 16 slides had a parasite density <500 p/*μ*l and 3 slides were <1000 p/*μ*l. On the basis of accurate qualitative identification, when further classifying whether it is positive of *P. falciparum*, 34 (55.7%) representatives got all correct results, but 5 (8.2%) and 22 (36.1%) representatives were wrong with 2 and 1 pieces, and the *P. falciparum*-positive slides were more likely to be classified as other non-*Plasmodium falciparum* species (*P* = 0.044, Fisher's exact test) (Table 6).

A total of 38 (27.3%) representatives from county-level medical institutions correctly identified 5 blood films, and 61 (43.9%), 29 (20.9%), 10 (7.2%), and 1 (0.7%) representatives correctly answered 4, 3, 2, and 1 blood films, respectively. In terms of qualitative identification, 84 (60.4%) of them got correct results of all 5 slides, and 2 (1.4%), 6 (4.3%), and 47 (33.8%) were wrong with 3, 2, and 1 slides, respectively. Moreover, the *Plasmodium*-negative and *P. falciparum*-positive slides were more likely to be misjudged (*P* < 0.001, Fisher's exact test) (Table 7). In addition to 26 negative slides that were wrongly classified, a total of 35 *P. falciparum*-positive slides were identified as negative, of which 33 slides were <500 p/*μ*l, 1 slide < 1000 p/*μ*l, and 1 slide > 10000 p/*μ*l; one *P. malariae*-positive slide was identified as negative, with the parasite density of 1731 p/*μ*l; three *P. vivax*-positive slides were identified as negative, and the parasite density was <500 p/*μ*l each. On the basis of qualitative accuracy, when further identify whether it is *P. falciparum*, a total of 70 (50.4%) representatives got all correct results, but 2 (1.4%), 15 (10.8%), and 52 (37.4%) representatives answered 3, 2, and 1 slides wrong, respectively, and the *P. falciparum*-positive slides were also more likely to be classified as other non-*Plasmodium falciparum* species (*P* < 0.001, Fisher's exact test) (Table 8).

### 3.3. Comparison of Malaria Microscopy Performance between CDCs/IPDs and Medical Institutions at Different Levels

In terms of qualitative identification, the accuracy among the representatives from CDCs/IPDs and medical institutions at provincial and county levels was not significantly different (*χ*^2^ = 5.519, *P* = 0.137) (Tables 1, 3, 5, and 7). Furthermore, no significance was found in accuracy of species identification among representatives from CDCs/IPDs and medical institutions at the provincial level (*χ*^2^ = 0.860, *P* = 0.407) (Tables 2 and 4). In addition, no significance was found in the accuracy of identification of *P. falciparum* or other non-*Plasmodium falciparum* species among representatives from CDCs and medical institutions at the county level (*χ*^2^ = 0.995, *P* = 0.330) (Tables 6 and 8).

## 4. Discussion

In the present study, the malaria microscopy competency in the counties and provinces being assessed in the subnational verification was analyzed. As a result, the diagnostic accuracy of malaria qualitative microscopy from provincial and county-level medical and CDC representatives was higher than 90%, respectively, with no statistical significance, but the slides with low parasite density (<500 p/*μ*l) were more likely to be misdiagnosed as negative. Moreover, the accuracy of species identification was from 80.0% to 85.0%, while the mutual misjudgment between *P. vivax* and *P. ovale* may be more likely to occur in CDCs/IPDs than that in medical institutes at provincial level, and the *P. falciparum*-positive slides were more likely to be classified as other non-*Plasmodium falciparum* species in medical institutions than that in CDCs at the county level.

Malaria is usually suspected first based on clinical symptoms, especially fever, but most cases of fever are usually not due to malaria, resulting in a high number of false-positive results and corresponding misuse of antimalarial drugs [15]. Parasitological testing is the only way to diagnose malaria accurately in febrile patients, and precise and prompt diagnosis with appropriate treatment is a key strategy to eliminate malaria [16]. It is much more important in the eliminating areas where the malaria incidence is approaching to zero and the very low chance of getting a fever from malaria. Therefore, China has continuously improved the case-based surveillance and response system and the malaria diagnosis laboratory network to ensure the testing competency of malaria parasites in CDCs/IPDs and medical institutions at all levels and provided strong technical support for malaria elimination [9]. The accuracy of more than 90% of qualitative identification and more than 80% of species identification found in this study, combined with all reported cases must be reviewed by malaria diagnosis reference laboratories through microscopy and nucleic acid testing [9, 12], convinces the fact that no indigenous malaria case has been reported since 2017 in China [17], and China has successfully eliminated this disease [14, 18].

However, the elimination of malaria is not the absence of malaria. China still faces the risk of malaria reestablishment of transmission [19–21], especially there are still thousands of imported cases every year [22], and there are still malaria-transmitting *Anopheles* mosquitoes, etc. [23]. Moreover, precise identification of slides with low parasite density is still a challenge in malaria microscopy in China [24]. On the other hand, low-density asymptomatic infections contribute far more to the malaria reservoir than previously thought and may be one of the main factors for the continuous spread of malaria [25, 26]. Thus, it is more important to timely and accurately identify malaria parasites in the post-elimination phase especially in the areas with risk of reestablishment transmission. In addition, accurate species identification, especially between *P. vivax* and *P. ovale*, has long been a bottleneck in microscopy [11, 12, 27, 28]. Meanwhile, long-term attention still needs to be paid to the timely and accurate identification of *P. falciparum*, which was the most common species among imported cases [29–31] and main cause of malaria death in China [32], so as to avoid misdiagnosis leading to the seriousness of the disease and even death. Therefore, it is necessary to maintain and further strengthen the sensitivity of malaria surveillance and the response system to prevent malaria reestablishment through improving the detection competency of malaria parasites in health institutions at all levels [23, 33], and the *P. falciparum* and *P. vivax* were the main species were assessed in the assessment.

First, given that the already existence of the sustained infrastructure, competent microscopists, good equipment and reagents and an appropriate workload, malaria microscopy remains the only inexpensive, easily used test for characterizing the presence of malaria parasites, identifying the *Plasmodium* species and quantifying parasite load [7], although it is not sensitive for detecting low-density parasitaemia, and not specific for identifying the species with similar morphology. Moreover, the microscopy competency is required in counties at risk of reestablishment of transmission according to the Technical Guideline for the Prevention of Malaria Re-Establishment in China. Second, it is recommended to promote the application of rapid diagnostic tests (RDT) to make up for the insufficient microscopy in some areas [34], although the current RDT kits are mainly histidine-rich protein 2-based for *P. falciparum* and lactate dehydrogenase-based for *Plasmodium* spp. Therefore, it is necessary to strengthen the development of kits that can identify different *Plasmodium* spp., while continuously improving their detection limits [35–37]. Third, we continue to strengthen laboratory network and actively promote nucleic acid detection methods for malaria parasites with the help of the nucleic acid testing laboratory platform at different levels established under the COVID-19 pandemic [38] and improve the sensitivity and specificity of malaria parasite detection from the front line. At the same time, it is necessary to develop more sensitive, specific, and rapid detection technologies or kits suitable for clinical use [35, 39].

## 5. Limitations

There are still some shortcomings in the present study, which needs to be further improved in the quality control of malaria microscopy in the future. First, in the preparation of blood slides, we try to use slides with the same specifications for the competency assessment of different microscopists in different regions, so that the results are completely comparable. Second, regarding the number of blood slides to be assessed, it is recommended to use more slides refer to the WHO ECA model to better reflect the competency of microscopists. Third, the selection of microscopists should be randomly selected blindly, so as to evaluate the actual capacity more objectively.

## 6. Conclusion

Malaria is still one of the most infectious diseases that cause a large number of cases and deaths, although there are more and more countries certified as malaria-free or approaching malaria elimination. A fully functional case-based surveillance system with qualified competency of case confirmation and classification to detect malaria infections that can prevent reestablishment of indigenous transmission in place is one of the necessary prerequisites for certification of a country's malaria-free status. Before WHO certification, an assessment on malaria microscopy competency in the counties and provinces was conducted in the subnational verification of malaria elimination in China, and qualified malaria microscopy competency was found, while some deficiencies, such as the identification of slides with low parasite density and more accurate species identification of *Plasmodium* spp., still needs to be improved. It is recommended to improve the timely and accurate identification of malaria parasites through continuous diagnostic platform construction, continuous technological innovation, and targeted training, so as to effectively support the updated strategy for the prevention of reestablishment, maintain achievement of malaria elimination, and reduce malaria deaths in China.

## Figures and Tables

**Table 1:** Qualitative identification based on malaria microscopy by representatives from provincial CDCs/IPDs.

Results	*Qualitative identification*					Total
	Negative	*P. falciparum*	*P. malariae*	*P. ovale*	*P. vivax*	
Correct	61	130	13	3	74	281
Wrong	9	7	0	0	3	19
Total	70	137	13	3	77	300

**Table 2:** Species identification of the *Plasmodium*-positive slides correctly in the qualitative microscopy by representatives from provincial CDCs/IPDs.

Correct answer	*Species identification*				Total
	*P. falciparum*	*P. malariae*	*P. ovale*	*P. vivax*	
*P. falciparum*	113	2	8	7	130
*P. malariae*	1	10	1	1	13
*P. ovale*	0	1	2	0	3
*P. vivax*	1	2	12	59	74

**Table 3:** Qualitative identification based on malaria microscopy by provincial-level representatives from medical institutes.

Results	*Qualitative identification*					Total
	Negative	*P. falciparum*	*P. malariae*	*P. ovale*	*P. vivax*	
Correct	40	88	12	3	46	189
Wrong	5	6	0	0	0	11
Total	45	94	12	3	46	200

**Table 4:** Species identification of the *Plasmodium*-positive slides correctly in the qualitative microscopy by provincial-level representatives from medical institutes.

Correct answer	*Species identification*				Total
	*P. falciparum*	*P. malariae*	*P. ovale*	*P. vivax*	
*P. falciparum*	69	5	10	4	88
*P. malariae*	0	10	1	1	12
*P. ovale*	0	0	3	0	3
*P. vivax*	2	3	4	37	46

**Table 5:** Qualitative identification based on malaria microscopy by representatives from county CDCs.

Results	*Qualitative identification*					Total
	Negative	*P. falciparum*	*P. malariae*	*P. ovale*	*P. vivax*	
Correct	61	116	16	7	75	275
Wrong	11	19	0	0	0	30
Total	72	135	16	7	75	305

**Table 6:** Identification of *P. falciparum* or not among the *Plasmodium*-positive slides correctly in the qualitative microscopy by representatives from county CDCs.

Correct answer	*Species identification*		Total
	*P. falciparum*	Non-*P. falciparum*	
*P. falciparum*	92	24	116
*P. malariae*	0	16	16
*P. ovale*	1	6	7
*P. vivax*	7	68	75

**Table 7:** Qualitative identification based on malaria microscopy by county-level representatives from medical institutes.

Results	*Qualitative identification*					Total
	Negative	*P. falciparum*	*P. malariae*	*P. ovale*	*P. vivax*	
Correct	142	277	31	20	160	630
Wrong	26	35	1	0	3	65
Total	168	312	32	20	163	695

**Table 8:** Identification of *P. falciparum* or not among the *Plasmodium*-positive slides correctly in the qualitative microscopy by county-level representatives from medical institutes.

Correct answer	*Species identification*		Total
	*P. falciparum*	Non-*P. falciparum*	
*P. falciparum*	201	76	277
*P. malariae*	1	30	31
*P. ovale*	4	16	20
*P. vivax*	7	153	160

## Data Availability

The data used to support the findings of this study are available from the corresponding author upon request.

## References

[1] WHO (2021). *World Malaria Report 2021*.

[2] WHO (2021). *Zeroing in On Malaria Elimination: Final Report of the E-2020 Initiative*.

[3] World Malaria Day (2025). *WHO launches effort to stamp out malaria in 25 more countries by 2025*.

[4] WHO (2020). *Preparing for Certification of Malaria Elimination*.

[5] WHO (2012). *Disease Surveillance for Malaria Elimination: An Operational Manual*.

[6] WHO (2017). *A Framework for Malaria Elimination*.

[7] WHO (2015). *Malaria Microscopy Quality Assurance Manual—Version 2*.

[8] WHO (2014). WHO procedures for certification of malaria elimination. *Releve Epidemiologique Hebdomadaire*.

[9] Yin J., Li M., Yan H., Zhou S., Xia Z. (2022). Laboratory diagnosis for malaria in the elimination phase in China: efforts and challenges. *Frontiers of Medicine*.

[10] Li M., Zhou H., Yan H. (2019). Analysis on external competency assessment for malaria microscopists in China. *Malaria Journal*.

[11] Yin J., Yan H., Li M. (2017). Competency and challenges in malaria microscopy in China. *Bioscience Trends*.

[12] Yin J. H., Yan H., Huang F. (2015). Establishing a China malaria diagnosis reference laboratory network for malaria elimination. *Malaria Journal*.

[13] Wang Q., Xu J., Hao Y. (2019). Assessment on the diagnostic capacity for parasitic diseases of health facilities—China. *China CDC Weekly*.

[14] *From 30 Million Cases to Zero: China Is Certified Malaria-Free by WHO*.

[15] WHO (2011). *Universal Access to Malaria Diagnostic Testing: An Operational Manual*.

[16] Landier J., Parker D. M., Thu A. M. (2016). The role of early detection and treatment in malaria elimination. *Malaria Journal*.

[17] Zhang L., Feng J., Zhang S. S., Xia Z. G., Zhou S. S. (2018). The progress of national malaria elimination and epidemiological characteristics of malaria in China in 2017. *Chinese Journal of Parasitology & Parasitic Diseases*.

[18] Feng J., Zhang L., Xia Z. G., Zhou S. S., Xiao N. (2021). Malaria-free certification in China: achievements and lessons learned from the national malaria elimination programme. *Zoonoses*.

[19] Sun Y. W., Yu D. M., Chen J. (2017). Two individual incidences of vivax malaria in Dandong municipality of Liaoning province. *Chinese Journal of Public Health*.

[20] Wang D., Li S., Cheng Z. (2015). Transmission risk from imported Plasmodium vivax malaria in the China-Myanmar border region. *Emerging Infectious Diseases*.

[21] Zhang L., Feng J., Zhang S. S., Xia Z. G., Zhou S. S. (2019). Epidemiological characteristics of malaria and the progress towards its elimination in China in 2018. *Chinese Journal of Parasitology & Parasitic Diseases*.

[22] Zhang S. S., Feng J., Zhang L. (2019). Imported malaria cases in former endemic and non-malaria endemic areas in China: are there differences in case profile and time to response?. *Infectious diseases of poverty*.

[23] Yin J., Yan H., Li M. (2022). Prompt and precise identification of various sources of infection in response to the prevention of malaria re-establishment in China. *Infectious Diseases of Poverty*.

[24] Huang F., Takala-Harrison S., Liu H. (2017). Prevalence of clinical and subclinical Plasmodium falciparum and Plasmodium vivax malaria in two remote rural communities on the Myanmar-China border. *The American Journal of Tropical Medicine and Hygiene*.

[25] Cheaveau J., Mogollon D. C., Mohon M. A. N., Golassa L., Yewhalaw D., Pillai D. R. (2019). Asymptomatic malaria in the clinical and public health context. *Expert Review of Anti-Infective Therapy*.

[26] Okell L. C., Bousema T., Griffin J. T., Ouedraogo A. L., Ghani A. C., Drakeley C. J. (2012). Factors determining the occurrence of submicroscopic malaria infections and their relevance for control. *Nature Communications*.

[27] Yin J., Zhang L., Feng J., Zhou S., Xia Z. (2020). Malaria diagnosis and verification—China, 2017-2018. *China CDC Weekly*.

[28] Li W., Zhang T., Xu X. (2020). Problems associated with the diagnosis of imported malaria in Anhui province, China. *The American Journal of Tropical Medicine and Hygiene*.

[29] Feng J., Tu H., Zhang L., Xia Z., Zhou S. (2020). Imported malaria cases—China, 2012–2018. *China CDC Weekly*.

[30] Zhang L., Feng J., Tu H., Yin J. H., Xia Z. G. (2021). Malaria epidemiological in China in 2020. *Chinese Journal of Parasitology & Parasitic Diseases*.

[31] Zhang L., Yi B. Y., Xia Z. G., Yin J. H. (2022). Epidemiological characteristics of malaria in China in 2021. *Chinese Journal of Parasitology & Parasitic Diseases*.

[32] Zhang L., Tu H., Zhou S., Xia Z., Feng J. (2021). Malaria deaths—China, 2011–2020. *China CDC Weekly*.

[33] Ding G., Zhu G., Cao C. (2018). The challenge of maintaining microscopist capacity at basic levels for malaria elimination in Jiangsu Province, China. *BMC Public Health*.

[34] Gunasekera W. D. A. W., Premaratne R. G., Weerasena O., Premawansa W. S., Handunnetti S. M., Fernando S. D. (2018). Utility of pf/pan RDT for diagnosis in the prevention of re-establishment of malaria in Sri Lanka. *Pathogens and Global Health*.

[35] The malERA Consultative Group on Diagnoses and Diagnostics (2011). Diagnostics: a research agenda for malaria eradication: diagnoses and diagnostics. *PLoS Medicine*.

[36] Acquah F. K., Donu D., Obboh E. K. (2021). Diagnostic performance of an ultrasensitive HRP2-based malaria rapid diagnostic test kit used in surveys of afebrile people living in Southern Ghana. *Malaria Journal*.

[37] Zainabadi K. (2021). Ultrasensitive diagnostics for low-density asymptomatic Plasmodium falciparum infections in low-transmission settings. *Journal of Clinical Microbiology*.

[38] *Notice on the Work Plan for Further Promoting the Capacity Building of the COVID-19 Nucleic Acid Amplification Testing Issued by the State Council’s Joint Prevention and Control Mechanism in Response to the COVID-19*.

[39] Kamaliddin C., Sutherland C. J., Houze S. (2021). The role of ultrasensitive molecular methods for detecting malaria-the broader perspective. *Clinical Infectious Diseases*.

